# Prognostic value of final pathological stage in colon adenocarcinoma after neoadjuvant chemotherapy: A propensity score-matched study

**DOI:** 10.3389/fsurg.2022.1022025

**Published:** 2022-10-26

**Authors:** Meijuan Xiang, Zongyu Liang, Yuan Gao, Xingyu Feng, Xueqing Yao

**Affiliations:** ^1^ School of Medicine, South China University of Technology, Guangzhou, China; ^2^ Department of Gastrointestinal Surgery, Department of General Surgery, Guangdong Provincial People's Hospital, Guangdong Academy of Medical Sciences, Guangzhou, China; ^3^ Department of General Surgery, Guangdong Provincial People's Hospital Ganzhou Hospital (Ganzhou Municipal Hospital), Ganzhou, China; ^4^ Department of Anorectal Surgery, Foresea Life Insurance Shaoguan Hospital, Shaoguan, China; ^5^ Second Department of General Surgery, The Sixth Affiliated Hospital, School of Medicine, South China University of Technology, Foshan, China; ^6^ The Second School of Clinical Medicine, Southern Medical University, Guangzhou, China; ^7^ The Fifth School of Clinical Medicine, Gannan Medical University, Ganzhou, China

**Keywords:** prognostic value, neoadjuvant chemotherapy, colon cancer, yp stage, survival

## Abstract

**Background:**

Neoadjuvant chemotherapy (NAC) could improve local tumor control of locally advanced colon cancer (LACC), but the prognostic value of yp stage in colon cancer remains unknown. Here, we aimed to ascertain yp stage as an indicator for LACC prognosis after NAC.

**Methods:**

The data of patients diagnosed with colon adenocarcinoma between 2004 and 2015 were extracted from the Surveillance, Epidemiology, and End Results database. After 1:2 propensity score matching, cancer-specific survival (CSS) and overall survival (OS) were compared between the NAC and Non-NAC groups of different stage classifications. The correlation between clinical and pathological factors and CSS was identified.

**Results:**

A total of 49, 149, and 81 matched pairs of stage 0–I, II, and III patients, respectively, were generated for analysis. For stage 0–I (*p* = 0.011) and III (*p* = 0.015), only CSS in the NAC groups were inferior. Receiving NAC was an independent prognostic risk factor for patients with stage 0–I (hazard ratio, 7.70; 95% confidence interval, 1.820–32.5; *p* = 0.006) and stage III (hazard ratio, 1.73; 95% confidence interval, 1.11–2.68; *p* = 0.015).

**Conclusions:**

The CSS was poorer among LACC patients who underwent NAC than among those who did not. The yp stage of colon cancer after NAC has distinctive significance, which may contribute to predicting the prognosis and guiding the treatment of LACC patients after NAC.

## Introduction

Colon cancer, among the most common malignant tumors worldwide, accounted for approximately two-thirds of new colorectal cancer cases and deaths in 2020 (1, 2). Locally advanced colon cancer (LACC) patients are still routinely treated with up-front surgery followed by adjuvant chemotherapy. Although neoadjuvant therapy (NAT) is not yet standard, it is proven to improve the tumor downstaging and margin-negative resection rates in colon cancer, resulting in local tumor control and even pathological complete remission (PCR) without excess complications (3–8). In 2016, the NCCN guidelines added neoadjuvant chemotherapy (NAC) as an optional treatment for the clinical T4b colon cancer cohort (9). In regards to pathological stage (*p* stage), an important factor that affects prognosis, several studies have reported that the final pathological stage after NAT (yp stage) is a significant predictive factor of survival outcomes among patients with rectal cancer who underwent chemoradiotherapy (10, 11). However, the prognostic value of yp stage in colon cancer remains unknown. Thus, we retrospectively analyzed cancer-specific survival (CSS) and overall survival (OS) of colon cancer patients treated with or without NAC in the Surveillance, Epidemiology, and End Results (SEER) database through propensity score matching (PSM) analysis, aiming for evaluating the effectiveness of yp stage as a prognostic indicator and adjuvant treatment guideline for colon cancer.

## Material and methods

### Patient selection

The data of patients pathologically diagnosed with primary colon adenocarcinoma between January 1, 2004 and December 31, 2015 were extracted from the SEER database (SEER*Stat Version 8.3.9). The study inclusion criteria were as follows: (1) histological type limited to colon adenocarcinoma (International Classification of Diseases for Oncology, 3rd edition codes for adenocarcinoma: 8140/3, 8143/3, 8144/3, 8210/3, 8261/3, 8263/3, 8220/3, and 8221/3; special type adenocarcinoma: 8141/3,8211/3, 8213/3, 8255/3, 8260/3, 8262/3, 8310/3, 8323/3, 8440/3, 8460/3, 8470/3, 8480/3, 8481/3; and signet ring cell carcinoma: 8490/3); (2) non-metastatic colon cancer; (3) radical intestinal resection; and (4) receipt or non-receipt of NAC (regardless of whether he/she uses preoperative radiotherapy or not). Surgery and systemic therapy sequences were limited to systemic therapy before surgery, systemic therapy both before and after surgery, surgery both before and after systemic therapy, systemic therapy after surgery, and no systemic therapy and/or surgical procedures. Surgery and radiation sequences were limited to radiation before surgery and no radiation and/or cancer-directed surgery; and (5) accurate prognostic information. The study exclusion criteria were as follows: (1) non-primary or multiple primary cancers; (2) rectosigmoid-Junction cancer; (3) unknown American Joint Committee on Cancer (AJCC) 7th pathological stage; (4) surgery including local tumor resection, none, or unknown; and (5) patients without NAC did not receive the standard treatment which the NCCN guidelines recommended to each pathological stage. (6) survival time of 0.

### Variables collected

(1) Patient information: sex, age at diagnosis, year at diagnosis, race, and insurance; (2) Tumor information: primary site, tumor size (Ts), pathological grade, histological type, tumor-node-metastasis stage, number of lymph nodes (LNs) detected; (3) Treatment data: sequence of chemotherapy, whether preoperative radiotherapy was administered; (4) Follow-up data: CSS and OS. CSS was defined as the time interval between the diagnosis of colon cancer and death caused by colon cancer. OS was defined as the time interval between the diagnosis of colon cancer and death from any cause. All the included cases were re-staged by the AJCC 7th edition according to the data provided by the SEER database.

### Patient classification

In this study, patients whose systemic therapy sequence was recorded as systemic therapy before surgery, systemic therapy both before and after surgery, and surgery both before and after systemic therapy were classified into the NAC group, whereas those whose systemic therapy sequence was recorded as systemic therapy after surgery or no systemic therapy and/or surgical procedures were classified into the Non-NAC group.

### Statistical analysis

All data were sorted out and analyzed by R software (version 4.1.2). Continuous variables were compared using the unpaired t-test, while categorical variables were compared using the *χ*2 test. CSS curves were estimated using the Kaplan-Meier method and compared using the log-rank test. Factors associated with CSS were estimated by uni- and multivariate analyses using the Cox proportional hazards model. Factors with *p* < 0.05 in univariate analysis were included in multivariate analysis.

A 1:2 PSM analysis without replacement was conducted *via* the nearest neighbor method with a caliper of 0.1 times the standard deviation of the propensity score (12). Matched variables included: age, sex, race, insurance, primary site, tumor size, pathological grade, histological type, AJCC stage, and number of LNs detected. Two-sided values of *p* < 0.05 were considered statistically significant.

## Results

### Baseline characteristics

**Figure 1:**
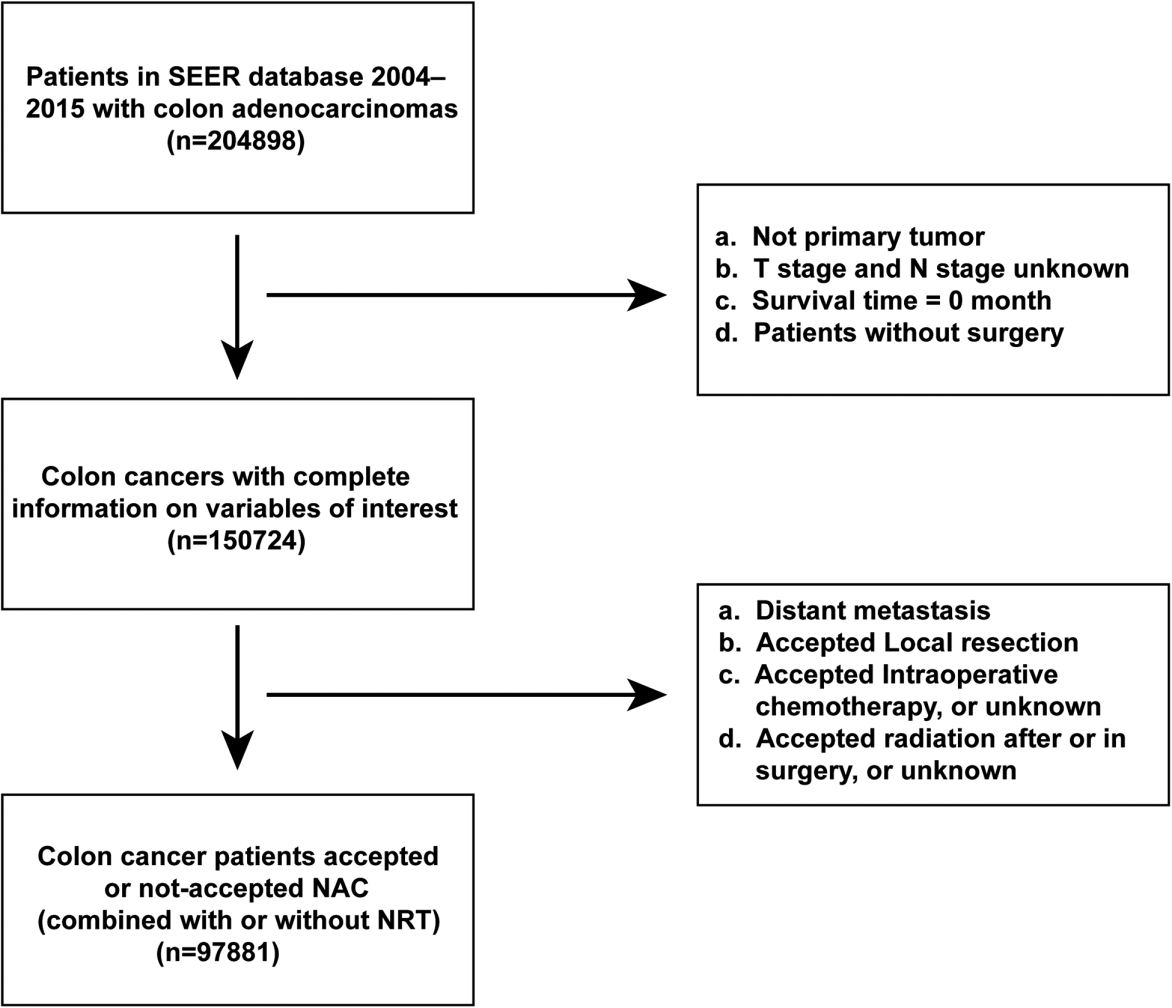
Flowchart of the patient selection process.

**Figure 2:**
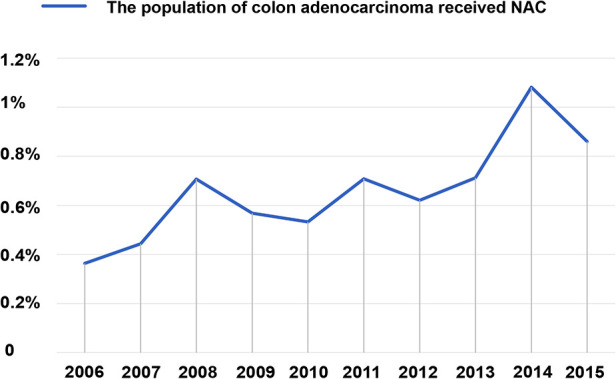
Rates of all patients with colon adenocarcinoma who received NAC were recorded in the SEER database from 2006 to 2015. The proportion received NAC in colon adenocarcinoma increased gradually from 3.635% in 2006 to 8.607% in 2015. NAC, neoadjuvant chemotherapy.

A total of 97,881 patients were included in this study (Figure 1). Of these, 458 patients underwent NAC, whereas 97,423 did not. Baseline demographic and clinicopathological characteristics were presented in Table 1. We found that the proportion of patients who received NAC for colon adenocarcinoma increased gradually from 3.635% in 2006 to 8.607% in 2015 (Figure 2). The median survival time was 66.0 [95% confidence interval (CI), 69.8–70.3] months in the Non-NAC group and 54.0 (95% CI, 58.8–65.6) months in the NAC group (*p* = 0.003; Table 1).

**Table 1:** Patient characteristics (*N* = 97,881).

Characteristics	Non-NAC group	NAC group	*p*-value
	(*N* = 97,423)	(*N* = 458)	
Age			<0.001
< 50 years	9,110 (9.4%)	88 (19.2%)	
≥ 50 years	88,313 (90.6%)	370 (80.8%)	
Sex			<0.001
Male	50,166 (51.5%)	183 (40.0%)	
Female	47,257 (48.5%)	275 (60.0%)	
Insurance			0.247
Yes	82,313 (84.5%)	397 (86.7%)	
No	15,102 (15.5%)	61 (13.3%)	
Unkown	8 (0%)	0 (0%)	
Race			0.629
White	75,272 (77.3%)	346 (75.5%)	
Black	13,987 (14.4%)	72 (15.7%)	
Others	8,164 (8.4%)	40 (8.7%)	
Location			<0.001
Left colon	61,456 (63.1%)	194 (42.4%)	
Right colon	35,967 (36.9%)	264 (57.6%)	
Differentiation Grade			<0.001
Grade 1–2	76,442 (78.5%)	324 (70.7%)	
Grade 3–4	16,983 (17.4%)	75 (16.4%)	
Unkown	3,998 (4.1%)	59 (12.9%)	
Histology type			0.124
Adenocarcinoma	87,322 (89.6%)	398 (86.9%)	
Special type adenocarcinoma	9,173 (9.4%)	56 (12.2%)	
Signet ring cell carcinoma	928 (1.0%)	4 (0.9%)	
Pathological stage			<0.001
Stage 0–I	28,349 (29.1%)	49 (10.7%)	
Stage II	34,949 (35.9%)	160 (34.9%)	
Stage III	34,125 (35.0%)	249 (54.4%)	
T-stage			<0.001
T0–2	32,768 (33.6%)	79 (17.2%)	
T3–4	64,655 (66.4%)	379 (82.8%)	
N-stage			
N0	63,298 (65.0%)	209 (45.6%)	<0.001
N1	22,515 (23.1%)	181 (39.5%)	
N2	11,610 (11.9%)	68 (14.8%)	
LNs-examined			<0.001
<12	18,286 (18.8%)	124 (27.1%)	
≥12	78,781 (80.9%)	327 (71.4%)	
Unkown	356 (0.4%)	7 (1.5%)	
Tumor size			<0.001
<4	38,306 (39.3%)	135 (29.5%)	
≥4	59,117 (60.7%)	323 (70.5%)	
PCT			0.500
Non-PCT	69,735 (71.6%)	321 (70.1%)	
PCT	27,688 (28.4%)	137 (29.9%)	
Survival time (OS)			
Median survival time (95%CI)	66.0 (69.8, 70.3)	54.0 (58.8, 65.6)	0.003

CI, confidence interval; LNs-examined, number of lymph nodes examined; PCT, postoperative chemotherapy.

### Propensity score matching

To minimize confounding factors, we respectively matched the Non-NAC and NAC groups in stage 0–I, II, and III cohorts to achieve a balanced distribution of these baseline covariates between the paired groups. As a result, yp stage 0–I (*n* = 49) and *p* stage 0–I (*n* = 98), 149 yp stage II (*n* = 149) and *p* stage II (*n* = 295), and 81 yp stage III (*n* = 81) and *p* stage III (*n* = 160) were matched (Supplementary Table S1).

For analysis of subgroups, we also matched the *p* stage 0–I group with the postoperative chemotherapy (PCT) subgroup (*n* = 10) and the non-PCT subgroup (*n* = 39) in the yp 0–I group separately. Moreover, the PCT subgroup (*n* = 4) and non-PCT subgroup (*n* = 6) of the yp stage 0–I group were also subjected to PSM matching. In stage II, non-PCT cohort in NAC group (*n* = 116) PSM matched with non-PCT cohort in Non-NAC group (*n* = 229), while PCT cohort in NAC group (*n* = 33) matched with PCT cohort in Non-NAC group (*n* = 66). The patient and tumor characteristics were well-balanced between the matched cohorts (*p* > 0.05).

### CSS and OS stratified by preoperative therapy

**Figure 3:**
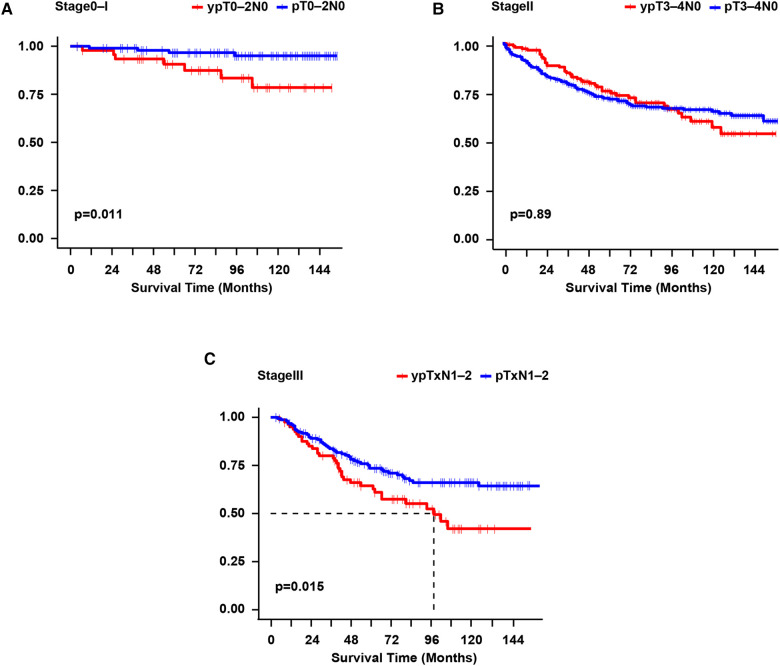
Survival curves were constructed per the Kaplan-Meier method for cause-specific survival for each pathological stage. Log-rank test for *p*-value. (**A**): yp stage 0-I vs. *p* stage 0-I; (**B**): yp stage II vs. *p* stage II; (**C**): yp stage III vs. *p* stage III. yp, the final pathological stage after neoadjuvant chemotherapy; *p*, pathological stage.

Among stage 0–I patients, CSS was significantly poorer in the NAC group than in the Non-NAC group (*p* = 0.011) (Figure 3A). Interesting, the CSS of the matched NAC -PCT group (*n* = 10) and Non-NAC group (*n* = 20) was similar (*p* = 0.140) (Supplementary Figure S1A). However, the NAC -non-PCT group (*n* = 39) had significantly worse CSS than the Non-NAC group (*n* = 78) (*p* = 0.012) (Supplementary Figure S1B). Moreover, the 5-year CSS was 50% in the matched Non-NAC -PCT group (*n* = 4) vs. 33.3% in the matched Non-NAC -non-PCT group (*n* = 6), the difference of which was not statistically significant on the univariate log-rank test (*p* = 0.410) (Supplementary Figure S1C). Among stage II patients, there was no significant difference in CSS (*p* = 0.890) between patients who received NAC and those who did not (Figure 3B). Additionally, the stratified analysis showed that the CSS of the NAC -non-PCT group (*n* = 116) was similar to that of the Non-NAC -non-PCT group (*n* = 229) (*p* = 0.650) (Supplementary Figure S1D). Consistently, the CSS of patients who received PCT (including T4b) in the yp stage II group (*n* = 33) was similar to that of patients in the *p* stage II group (*n* = 66) (*p* = 0.660) (Supplementary Figures S1E,F). Moreover, among the stage III patients, the CSS was significantly worse for the NAC vs. Non-NAC group (*p* = 0.015) (Figure 3C). Nevertheless, there was no significant difference in OS among the stage 0–I (*p* = 0.870), stage II (*p* = 0.074), and stage III groups (*p* = 0.130) groups (Supplementary Figure 2).

### Prognostic factors for CSS of different stage classifications

As shown in Supplementary Table S2, uni- and multivariate Cox analyses demonstrated that, in the NAC group, not receiving PCT (hazard ratio [HR], 7.70; 95% confidence interval [CI], 1.820–32.500; *p* = 0.006) was an independent prognostic risk factor of CSS for patients. In the matched stage 0-I groups. In the matched stage II groups, age ≥ 50 years (HR, 2.436; 95% CI, 1.350–5.225; *p* = 0.011), T4a stage (HR, 3.065; 95% CI, 1.779–5.282; *p* < 0.001), T4b stage (HR,3.065; 95% CI, 2.308–5.110; *p* < 0.001) and poor histological differentiation (HR, 1.971; 95% CI, 1.244–3.123; *p* = 0.004) were independent risk factors, while ≥12 detected LNs (HR, 0.38; 95% CI, 0.326–0.652; *p* < 0.001) was an independent protective factor for CSS. In the matched stage III groups, only receiving NAC (HR, 1.657; 95% CI, 1.069–2.631; *p* = 0.024) was independently and significantly associated correlated with CSS.

## Discussion

Due to the advantages of NAC of improving tumor downstaging, improving the R0 resection rate, and even prolonging disease-free survival time, its application in LACC is gradually increasing (3, 4, 6, 7, 13–15). Nonetheless, it remains unknown whether the final pathological stage (yp stage) of LACC patients who received NAC has a similar prognostic value to that of the usual postoperative pathological stage (*p* stage). To our knowledge, this is the first study to focus on the prognostic significance of yp stage in LACC patients after NAC. As the large difference in sample size between the NAC and Non-NAC groups, we balanced the clinical characteristics of the two groups using PSM for more reliable results. With the PSM and analysis of the survival time among stages 0–I, II, and III, respectively, we found that the CSS of LACC patients who underwent NAC was poorer than that of patients at the same pathological stage who did not, which indicated that yp stage of colon adenocarcinoma after NAC has significantly prognostic value and may provide evidence for PCT following curative surgery.

The present study found that young patients, those with right colon cancer, and those with poorly differentiated cancer were more likely to receive NAC. The shorter survival time in the NAC group may be due to worse histological grade, fewer detected LNs, later TN stage, and greater tumor bulk burden in the NAC group. As reported, *p* stage I colon cancer has good prognosis after radical resection alone. Compared with pT1-2N0 patients who did not receive chemoradiotherapy, ypT1-2N0 patients who received NAT preparation were more likely to relapse, and the recurrence rates of ypT0N0 and ypT1-2N0 were 2.7% and 12.3%, respectively (16). Moreover, several studies reported that, compared with *p* stage I patients, those with yp stage I disease had many risk factors leading to poor outcomes, such as poorer tissue differentiation, later T stage, and higher carcinoembryonic antigen levels (17, 18).

As for the stage 0–I patients in our study, the NAC group showed an inferior CSS to the Non-NAC group, which was also consistent with previous reports in NAT among rectal malignant tumors. Furthermore, the stratified analysis showed that the CSS was significantly worse in the yp stage 0–I non-PCT vs. *p* stage 0–I group, while the CSS of the yp stage 0–I with PCT group was similar to that of the *p* stage0–I group. Consistently, the 5-year CSS of the yp stage 0–I with PCT group was better than that of the yp stage 0–I non-PCT group, but the difference was not statistically significant. These results suggested that patients with yp stage 0–I colon cancer may benefit from PCT, which supports the findings of Collette et al. regarding rectal cancer in that LARC patients downgraded to ypT0-2 after preoperative radiotherapy can benefit from PCT (19).Other recent studies also demonstrated that patients with rectal cancer who reached PCR after NAT also benefited from PCT (20, 21). Patients who achieved descending stage after NAT also responded to adjuvant treatment (19). PCT for patients who respond to NAT may be beneficial by potentially eradicating residual micrometastatic disease (19, 22). Therefore, receiving adjuvant chemotherapy may contribute to the prognosis of these patients. A further randomized trial with a larger sample size is warranted to compensate for the limited sample size, especially in the PCT and non-PCT groups, of yp stage 0–I patients in our study.

As for stage II, CSS and OS were similar in the NAC and Non-NAC groups. This finding suggests that the risk of yp stage II patients may be classifiable according to the same criteria as and receive the same treatment regimen for *p* stage II. However, there was wide heterogeneity among stage II colon cancer patients, and the 5-year OS ranged from 58.4% (IIc) to 87.5% (IIa). Therefore, stage II patients require stratification to distinguish between high- and low-risk groups and guide the choice of treatment. However, the relevant information was not available in the SEER database.

Our study also found that the CSS of yp stage III patients was significantly poorer than that of *p* stage III patients, aligned with previous researches that patients with N+ after chemotherapy had a poor prognosis (23, 24). However, it is difficult to determine whether patients with a poor NAT response will benefit from PCT (25–27). Collette et al. reported that patients with a poor NAC response (ypN2) could not benefit from PCT and that the poor response may indicate resistance to treatment (19). Thus, we inferred that patients with yp stage III colon cancer may require adjustment to the adjuvant chemotherapy regimen. As another study reported that patients with *p* stage III disease had a better survival rate than those with yp stage III rectal cancer, the recurrence-free survival rate of yp stage III patients was intended to increase after treatment with second-line chemotherapy (28). Notably, the loss of DNA mismatch repair protein expression may occur in fluorouracil-based chemotherapy-insensitive colon cancer patients (29). Therefore, combination treatment with immunotherapy may be a promising research direction to improve the prognosis of patients with yp stage III colon cancer.

We also identified some independent risk factors for CSS. Poor tumor differentiation was an independent risk factor for prognosis in stage II patients. Poorly differentiated and undifferentiated tumors are more likely to metastasize distantly, the main cause of tumor death. Another independent risk factor found for CSS in this research was T4 for stage IIpatients. Regardless of LN status, colon cancer with advanced local invasion (T4) is more prone to local recurrence and distant metastasis, resulting in a low survival rate. Besides, according to the American Society of Clinical Oncology and the European Society of Medical Oncology, the number of LNs dissected (<12) is a risk factor for recurrence in patients with stage II colorectal cancer, consistent with our study findings (30, 31).

Another finding was that CSS was poorer for patients who underwent NAC than for those who did not undergo NAC, while the OS was similar between the two groups. Acknowledgedly, CSS is only related to tumor death, while OS is related to death caused by any reason. Although we adjusted variables that may lead to OS differences, including age, gender, diagnosis year, etc., we did not balance many other potential confounders between the NAC group and Non-NAC group, such as basic disease and economic status. Therefore, we speculated that there was no difference in OS because more patients in the Non-NAC group died of other causes, thus offsetting the difference in CSS between the two groups. This retrospective study had possible selective bias despite the PSM analysis and the fact that we selected patients treated with standard therapy whenever possible. Also, the SEER database lacks several important characteristics, such as chemotherapy or radiotherapy dosage, mismatch repair/micro-satellite instability status, perineural invasion, and lymphatic vascular invasion. Therefore, we cannot adjust for these potential confounding factors, especially in stage II patients, which made it impossible to distinguish the low- and high-risk groups for a further stratified analysis. Thus, large-scale prospective randomized studies are needed that explore the prognostic value of the yp stage in LACC.

## Conclusions

The CSS was poorer for patients who underwent NAC than for those who did not undergo NAC in the same pathological stage, while the OS was similar between the two groups. Our results suggest that the final pathological stage of colon cancer after NAC has different clinical significance from the usual postoperative pathological stage and may be used to predict prognosis and guide treatment for LACC patients after NAC.

## Data Availability

Publicly available datasets were analyzed in this study. This data can be found here: https://seer.cancer.gov/.
