# Supplementary material for: Prognostic value of final pathological stage in colon adenocarcinoma after neoadjuvant chemotherapy: A propensity score-matched study
Source: Front Surg. 2022 Oct 26;9:1022025. doi: 10.3389/fsurg.2022.1022025 (PMC9643450; doi:10.3389/fsurg.2022.1022025)
Supplement: Supplementary file 1 [file Datasheet1.docx]

Supplementary Material

# Supplementary Figures and Tables

## Supplementary Figures

**Supplementary Figure 1:**


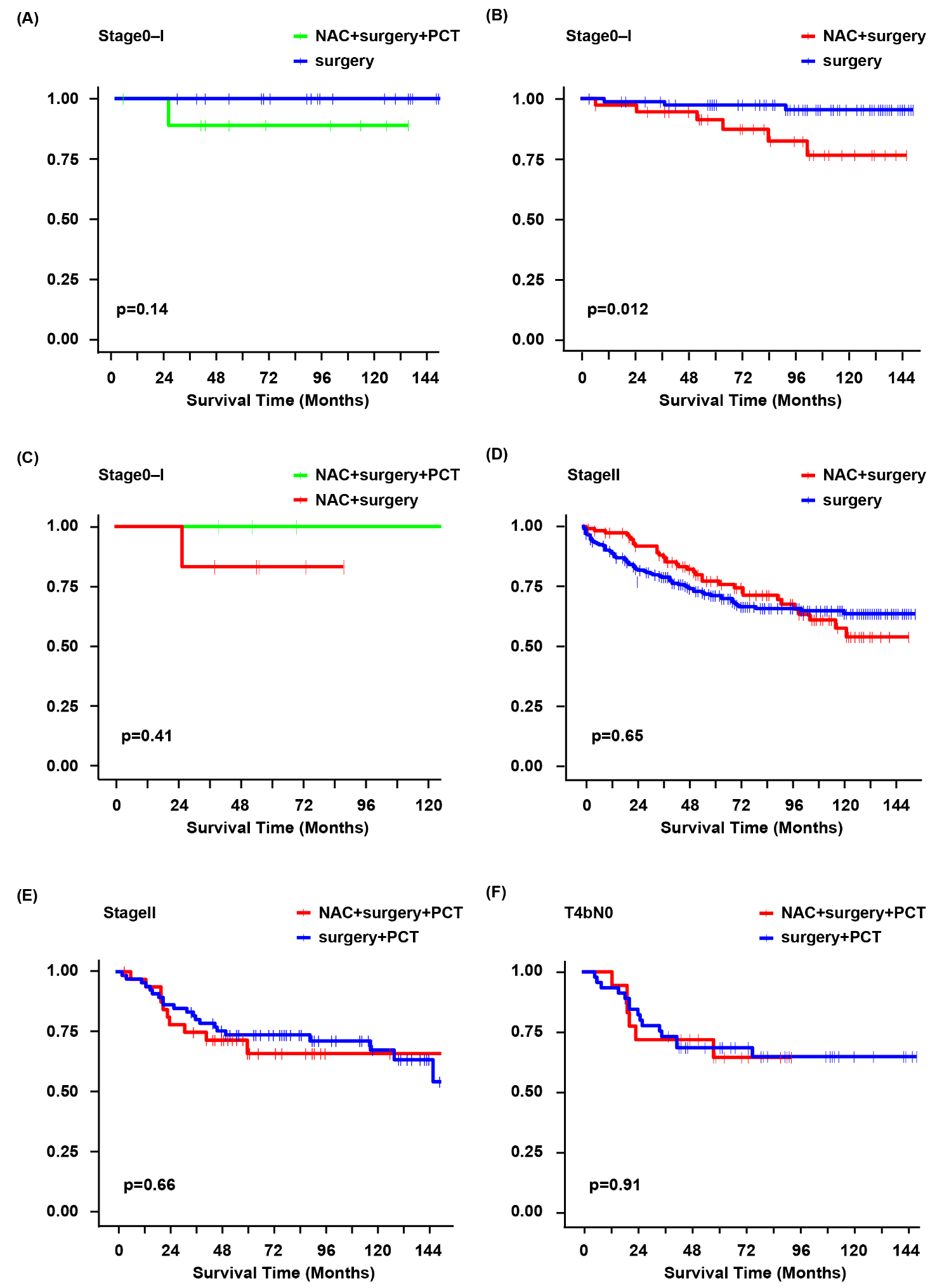


**Supplementary Figure 1.** Survival curves were constructed per the Kaplan-Meier method for cause-specific survival for each matched subgroup. Log-rank test for P-value. (A): yp stage 0–I disease with NAC, surgery, and PCT successively vs. p stage 0–I disease with surgery alone; (B): yp stage 0–I disease with NAC and surgery but without PCT vs. p stage 0–I disease with surgery alone; (C): yp stage 0–I disease with NAC, surgery, and PCT successively vs. yp stage 0–I disease with NAC and surgery but without PCT. (D): yp stage II disease with NAC and surgery but without PCT vs. p stage II disease with surgery alone; (E): yp stage II disease with NAC, surgery, and PCT successively vs. p stage II disease with surgery and PCT; (F): ypT4bN0 disease with NAC, surgery, and PCT successively vs. pT4bN0 disease with surgery and PCT. NAC, neoadjuvant chemotherapy; PCT, postoperative chemotherapy; yp, the final pathological stage after neoadjuvant chemotherapy; p, pathological stage.

**Supplementary Figure 2:**

**
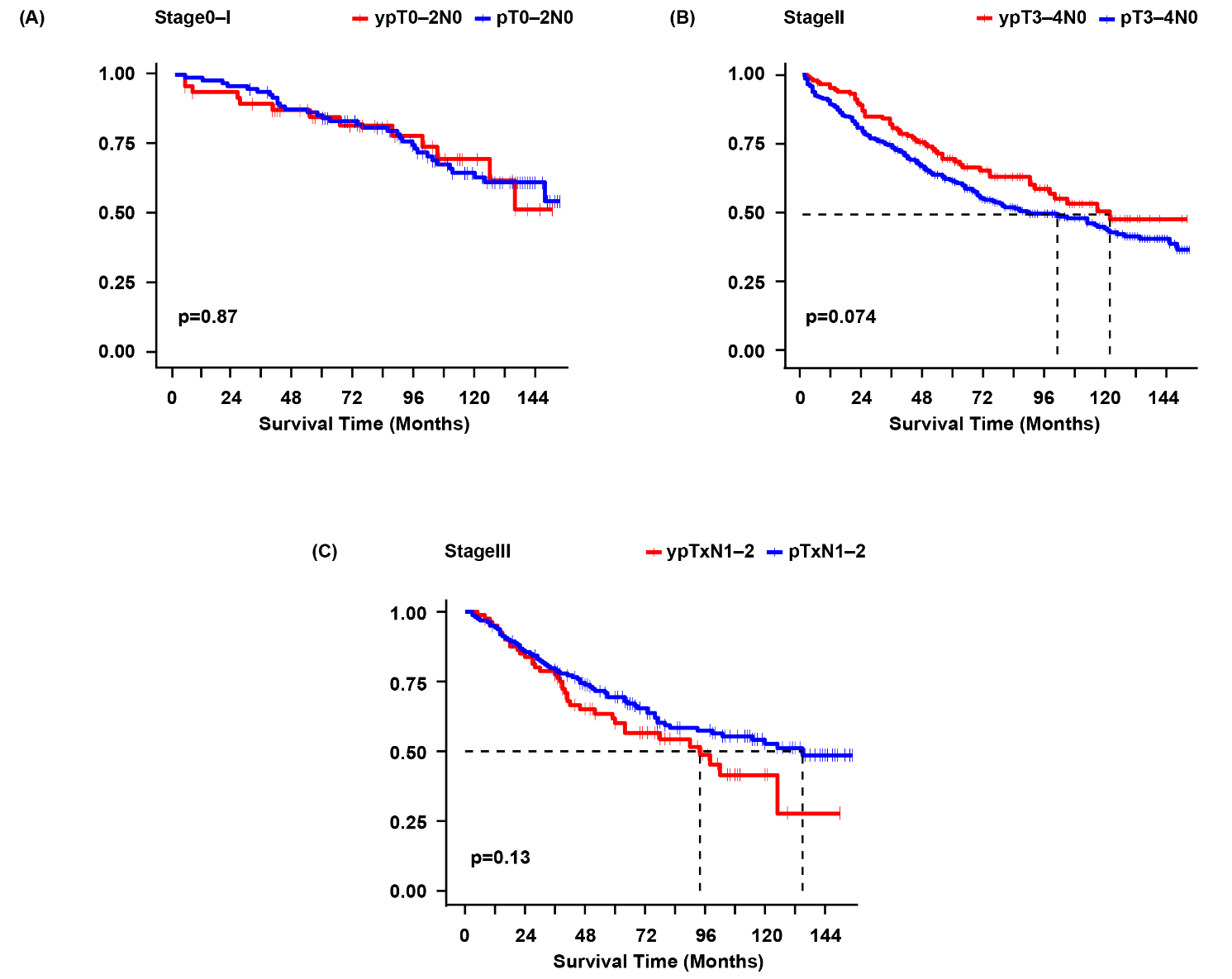
**

**Supplementary Figure 2.** Survival curves were constructed per the Kaplan-Meier method for overall survival for each pathological stage. Log-rank test for P-value. (A): yp stage 0–I vs. p stage 0-I; (B): yp stage II vs. p stage II; (C): yp stage III vs. p stage III. yp, the final pathological stage after neoadjuvant chemotherapy; p, pathological stage.

## Supplementary Table

**Supplementary Table 1.** Patient characteristics between NAC group and Non-NAC group at each stage before and after PSM

## Supplementary Table

**Supplementary Table 2.** Prognostic factors for CSS of different stage classifications
